# A comparative study of *Lachancea thermotolerans* fermentative performance under standardized wine production conditions

**DOI:** 10.1016/j.fochx.2024.101214

**Published:** 2024-02-09

**Authors:** Javier Vicente, Luka Vladic, Eva Navascués, Silvia Brezina, Antonio Santos, Fernando Calderón, Wendu Tesfaye, Domingo Marquina, Doris Rauhut, Santiago Benito

**Affiliations:** a Unit of Microbiology, Genetics, Biology Faculty, Physiology and Microbiology Department, Complutense University of Madrid, Ciudad Universitaria, S/N, 28040 Madrid, Spain; b Department of Food Science and Technology, University of Natural Resources and Life Sciences, Vienna, Gregor-Mendel-Straße 33, 1180 Wien, Austria; c Department of Microbiology and Biochemistry, Hochschule Geisenheim University (HGU), Von-Lade-Straße 1, 65366 Geisenheim, Germany; d Department of Chemistry and Food Technology, Polytechnic University of Madrid, Ciudad Universitaria, S/N, 28040 Madrid, Spain

**Keywords:** l-Lactic acid (PubChem CID107689), l-Malic acid (PubChem CID222656), Acetic acid (PubChem CID176), Succinic acid (PubChem CID1110), Isoamyl acetate (PubChem CID31276), Ethyl lactate (PubChem CID7344), Phenylethyl Alcohol (PubChem CID6054), *Lachancea thermotolerans*, l-lactic acid, Organic acids, Aroma compounds, Wine

## Abstract

The study explores diverse strains of *Lachancea thermotolerans* in single-inoculum wine fermentation conditions using synthetic grape must. It aims to analyze the role of the species without external influences like other microorganisms or natural grape must variability. Commercial strains and selected vineyard isolates, untested together previously, are assessed. The research evaluates volatile and non-volatile chemical compounds in final wine, revealing significant strain-based variations. *L. thermotolerans* notably produces lactic acid and consumes malic acid, exhibiting moderate ethanol levels. The volatile profile displays strain-specific impacts, affecting higher alcohol and ester concentrations compared to *S. cerevisiae*. These effects vary based on the specific compounds. Using a uniform synthetic must enables direct strain comparisons, eliminating grape-related, environmental, or timing variables in the experiment, facilitating clearer insights into the behavior of *L. thermotolerans* in wine fermentation. The study compares for the first time all available commercial strains of *L. thermotolerans*.

## Highlights

• For the first time all available commercial *L. thermotolerans* strains are compared.
• A synthetic reproducible grape juice is used to compare different yeast strains.
• The proposed comparative methodology is reproducible in any laboratory.
• The proposed methodology allows comparing yeast strains under the same conditions.
• Several chemical compounds are compared for the different studied strains.

## 1 Introduction

*Lachancea thermotolerans* is a non-*Saccharomyces* yeast that possesses the unique ability to significantly produce l-lactic acid under winemaking conditions (Jolly et al., 2003, Jolly et al., 2014, Jolly et al., 2017). This ability allows for efficient acidification of wines, increasing their total acidity and reducing the pH (Blanco et al., 2020, Porter et al., 2019a, Porter et al., 2019b, Vilela, 2018). Such increased acidity is of interest in warm wine regions that suffer from a lack of acidity, among other technical problems. The evolution of climate change can potentially increase the number of regions facing this problem, further increasing the interest in the use of *L. thermotolerans* (Benito, 2020).

The utilization of *L. thermotolerans* has emerged as the most reliable biological acidification strategy in winemaking (Vicente et al., 2022). While there are other effective technologies available to enhance wine acidity, the use of *L. thermotolerans* presents several notable advantages. Firstly, the stability of l-lactic acid surpasses that of other options involving chemical additions, such as the addition of unstable acids like tartaric acid, which precipitates upon combination with potassium ions, or malic and citric acids, which can be metabolized by lactic bacteria, leading to undesirable uncontrolled re-fermentations. Secondly, the implementation of *L. thermotolerans* does not require initial costly investments, unlike other technological alternatives such as inverse osmosis. Furthermore, commercial strains of *L. thermotolerans* can be readily obtained in any wine region at comparable prices to regular dehydrated *S. cerevisiae* commercial strains. Despite these advantages, the use of *L. thermotolerans*, like other non-*Saccharomyces* yeast species, does present certain concerns when employed on an industrial scale. These include its moderate fermentative power below 10 % (v/v) and sensitivity to sulfur dioxide (Vicente et al., 2021). Consequently, the available strains of *L. thermotolerans* should be employed in conjunction with a *S. cerevisiae* or *Schizosaccharomyces pombe* strain as these species are reported to be able to ferment over 15 % (v/v) (Benito, 2018), and applied to healthy grapes that do not require substantial additions of sulfur dioxide.

The first commercial strain of *L. thermotolerans*, known as ConcertoTM (Hansen), entered the market in 2012. Currently, there are seven strains of *L. thermotolerans* available in the market (Vejarano & Gil-Calderón, 2021): Concerto (CHR Hansen, Denmark), Laktia (Lallemand, Canada), Levulia Alcomeno (AEB Group, Italy), EnartisFerm QK (Enartis, Italy), Excellence (X’Fresh Lamothe-Abiet, France), Kluyveromyces thermotolerans (Probiotec, Italy), and Octave (CHR Hansen, Denmark). Furthermore, some manufacturers, such as CHR Hansen, have begun offering two different strains of *L. thermotolerans* with distinct purposes. This wide range of commercial options facilitates the acquisition of active freeze dried *L. thermotolerans* by winemakers worldwide, suitable for use in any winery regardless of the wine region. However, no previous study has compared all the commercially available strains of *L. thermotolerans* against each other.

There have been several previous studies comparing some of the available commercial strains of *L. thermotolerans*. Two studies compared the three commercial strains LevuliaTM (AEB), Concerto^TM^ (Hansen), and Laktia^TM^ (Lallemand). Another study compared three different commercial strains, namely Excellence X’Fresh (Lamothe-Abiet), Levulia^TM^ (AEB), and QKAPPA (Enartis) (Vicente et al., 2023a), while one study compared two strains, Laktia^TM^ (Lallemand) and Concerto^TM^ (Hansen) (Vaquero et al., 2020). These studies reported varying and sometimes contradictory results regarding the commercial strain that produces the highest concentration of l-lactic acid, with different orders reported in each study. Additionally, different chemical parameters exhibited different production rankings. This phenomenon can be attributed to the varying performance of each strain under different conditions, such as different combinations with *S. cerevisiae* strains or the use of different grape juices and fermentation conditions. None of these previous studies compared all the commercial strains under the same conditions in pure fermentation without combining them with *S. cerevisiae*.

This study evaluates the fermentative performance of several *L. thermotolerans* strains in a synthetic grape must (SGM) (Henschke and Jiranek, 1993). The proposed methodology enables the replication of the experiment in any laboratory, thereby facilitating the comparison of new future commercial *L. thermotolerans* strains or other selected strains under standardized conditions. The study encompasses all commercially available *L. thermotolerans* strains to date, as well as autochthonous strains isolated from Spanish vineyards and wineries.

### 1.1 Hypothesis

Given the varied and sometimes contradictory results from previous studies comparing different commercial strains of *Lachancea thermotolerans* (*L. thermotolerans*) in terms of l-lactic acid production under winemaking conditions, we hypothesize that there will be significant variability in fermentative performance among the commercially available *L. thermotolerans* strains and autochthonous strains isolated from Spanish vineyards and wineries. We anticipate observing distinct patterns of l-lactic acid production among these strains in synthetic grape must (SGM), reflecting the diverse genetic makeup and metabolic capabilities of each strain. Furthermore, we predict that the fermentation performance of these strains will differ when tested in pure fermentation without the addition of *Saccharomyces cerevisiae*, highlighting the importance of evaluating *L. thermotolerans* strains independently to understand their true potential for biological acidification in winemaking.

## 2 Material and methods

### 2.1 Microorganisms and fermentations

Thirteen strains of *Lachancea thermotolerans* yeast were utilized in this study: Concerto (CHR Hansen, Denmark), Laktia (Lallemand, Canada), Levulia Alcomeno (AEB Group, Italy), EnartisFerm QK (Enartis, Italy), Excellence (X'Fresh Lamothe-Abiet, France), and Octave (CHR Hansen, Denmark) as commercially available strains; and NG-108, A11-612, EM-119, MJ-311, BD-612, L1, and L3 (Complutense University of Madrid, Madrid, Spain) as selected strains. The control strain used was *S. cerevisiae* AWRI-796 (Maurivin, Australia). All *L. thermotolerans* strains employed in this study have previously been identified as different strains according to their genotype determined by the microsatellite typing protocol for *L. thermotolerans*, amplifying six different microsatellites by multiplexed PCR, and resolving them by agarose electrophoresis (Vicente et al., 2023c).

Synthetic Grape Must (SGM), prepared according to the original formulation (Henske and Jiranek, 1993), was utilized for all fermentations. Briefly, the SGM composition included 200 g/L of equimolar glucose and fructose, 3 g/L of malic acid, and 2.5 g/L of potassium tartrate, with pH adjusted to 3.5. The nitrogen content was adjusted to 140 mg/L from amino acids and 60 mg/L from di-ammonium phosphate. Yeast precultures were incubated in SGM at 25 °C and 150 rpm orbital shaking for 24 h. For the fermentations, conducted in triplicate, the final inoculation concentration was 2·10^5^ cells/mL in 100 mL borosilicate bottles containing 90 mL of SGM. The bottles were then incubated at 25 °C. Fermentative kinetics were monitored by measuring weight loss every 24 h, and fermentations were considered complete when the weight loss was less than 0.01 % per day. After fermentation, all wines were centrifuged (7000 rpm for 5 min) and stored at 4 °C until further analysis.

### 2.2 Basic oenological parameters determinations

A Y15 Autoanalyzer and its enzymatic kits (Biosystems, Spain) were used to perform determinations of l-malic acid and l-lactic acid. The determination of acetic acid, ethanol, total acidity, glucose + fructose, succinic acid, pH, and glycerol in the resulting wines was carried out using an FTIR autoanalyzer Bacchus 3 (TDI, Spain).

### 2.3 Volatile compounds

The analysis of esters, higher alcohols, and fatty acids was performed using headspace solid phase micro extraction in connection with gas chromatography coupled with mass spectrometry (HS-SPME-GC–MS) by the Department of Microbiology and Biochemistry at Hochschule Geisenheim University (Scansani et al., 2020, Jung et al., 2021). The analytical method is briefly described in the following:

1.7 g of sodium chloride (NaCl; p.a.) were weighted into a 20 mL amber glass headspace vial before 5 mL sample and 10 μL of each internal standard solution (1-octanol 600 mg/L (for quantification), cumene 52 mg/L (additionally for control)) were pipetted. Then the vial was tightly closed with a magnetic screw cap.

The calibration was performed in model wine (3 %, 6 % or 12 % (v/v) solutions of ethanol in water (depending on the ethanol content of the samples), 3 g/L tartaric acid, adjusted to pH 3). A multipurpose sampler MPS robotic (Gerstel, Mülheim an der Ruhr, Germany) was applied for HS-SPME injection. SPME extraction was conducted with a 1 cm SPME fiber with 65 μm of polydimethylsiloxane/ divinylbenzene (Supelco) for 20 min (incubation temperature: 40 °C, incubation time 10 min). The sample was transferred with a cooled injection system (CIS-4, Gerstel, Mülheim an der Ruhr, Germany) to the gas chromatograph (GC 7890 A, Agilent, Santa Clara, USA): temperature program: 30 °C (1 min), 12 °C/s to 240 °C (4 min); split ratio 1:10. The separation of the volatile compounds was achieved using a 60 m x 0.25 mm x1 μm gas chromatographic column (Rxi-5Sil, Restek, Bad Hom-burg, Germany) with the following oven program: 40 °C (4 min), with 5 °C /min to 210 ◦C and with 20 °C/min to 240 °C (10.5 min). Helium served as carrier gas (constant flow: 1.2 mL/min). Detection of the volatile substances was conducted with a mass spectrometer MS 5975B (Agilent, Santa Clara, USA) applying EI (70 eV) and scan mode (*m*/*z* 35–250). Instrumental control, acquisition of data and quantitative data analysis were carried out using Agilent MassHunter workstation software (Jung et al., 2021).

### 2.4 Statistical analyses

All statistical analyses were conducted using R software version 4.1.2 (R Development Core Team, 2013). Analysis of variance (ANOVA) and Tukey post-hoc tests were utilized to compare the different groups and values.

## 3 Results and discussion

### 3.1 Fermentative kinetic and main metabolites in the resulting wines

These three parameters—the fermentative kinetics, the residual sugars, and the final ethanol production—should be considered together since the fermentative kinetics were measured as weight loss (CO_2_ release from the alcoholic fermentation), which is directly proportional to the consumed sugars and the produced ethanol. Despite the fact that the sugar:ethanol ratio in this species is slightly altered due to the absence of a strong Crabtree effect and the conversion of some consumed sugars into lactic acid (Vicente et al., 2021), these parameters are closely related.

**Fig. 1:**
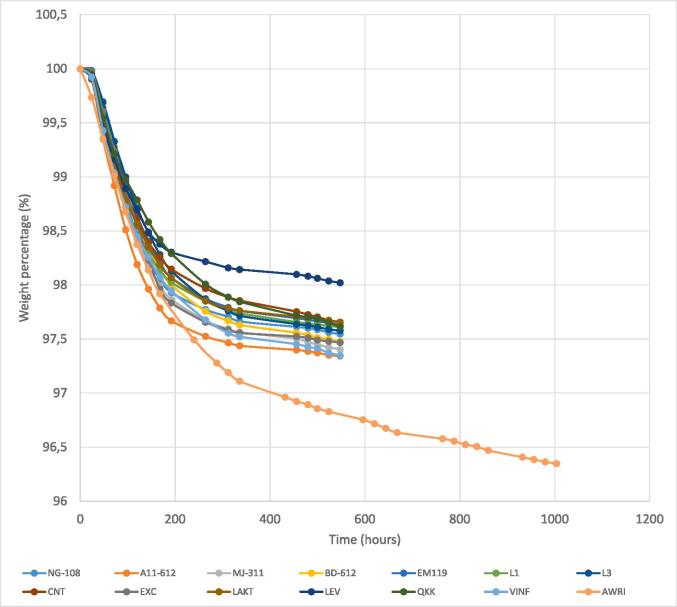
Fermentation kinetics of gravimetrically measured variants by total weight loss during pure fermentation in Synthetic Grape Must (SGM). The *Lachancea thermotolerans* strains used were CNT (Concerto, CHR Hansen, Denmark), LAKT (Laktia, Lallemand, Canada), LEV (Levulia Alcomeno, AEB Group, Italy), QKK (EnartisFerm QK, Enartis, Italy), EXC (Excellence, X'Fresh Lamothe-Abiet, France), and VINF (Octave, CHR Hansen, Denmark), along with the selected strains NG-108, A11-612, EM-119, MJ-311, BD-612, L1, and L3 (Complutense University of Madrid, Madrid, Spain). The *Saccharomyces cerevisiae* control strain used was AWRI (AWRI-796, Maurivin, Australia).

The fermentative kinetics of all the *L. thermotolerans* strains were different from that of the *S. cerevisiae* control, as well as the final residual sugars and ethanol concentration of the resulting wines. All *L. thermotolerans* strains exhibited a similar fermentative kinetics (Fig. 1), completing the fermentation in 23 days (three days longer than the *S. cerevisiae* control) and experiencing a weight loss of approximately 2.5 % (compared to the *S. cerevisiae* control, which had a weight loss of around 3.75 %). Only one strain, Levulia Alcomeno (LEV), exhibited a slower fermentation and a final weight loss of approximately 2.0 %.

These results, concerning the fermentative kinetics and the total amounts of released CO_2_, can be interpreted as an indirect estimation of the fermentative capacity of the strains under investigation. The final ethanol concentration ranged from 6.26 % to 8.59 %, whereas the *S. cerevisiae* control reached a significantly higher value of 11.26 % (v/v) (Table 1). The commercial strain Levulia exhibited the lowest ethanol concentration, while the autochthonous strain A11-612 showed the highest. Among the commercial strains, Excellence had the highest ethanol concentration at 8.12 % (v/v). The commercial strain Excellence along with the selected strains NG-108, A11-612, and MJ-311, exhibited significantly higher final ethanol concentrations compared to the commercial strains Levulia and Enartis. Strains EM-119, L1, L3, and the commercial strains Concerto and Laktia did not demonstrate statistically significant differences when compared to all the studied *L. thermotolerans* strains.

**Table 1:** Final chemical analysis of pure fermentations in Synthetic Grape Must (SGM). The *Lachancea thermotolerans* strains used were NG-108, A11-612, MJ-311, BD-612, EM-119, L1, L3 (Complutense University of Madrid, Madrid, Spain), Concerto (CHR Hansen, Denmark), Excellence (X'Fresh Lamothe-Abiet, France), Laktia (Lallemand, Canada), Levulia Alcomeno (AEB Group, Italy), EnartisFerm QK (Enartis, Italy) and Octave (CHR Hansen, Denmark). The *Saccharomyces cerevisiae* control strain used was AWRI-796 (Maurivin, Australia).

Strain	Ethanol (%)	pH	Total Acidity (g/L)	Acetic Acid (g/L)	Malic Acid (g/L)	Lactic Acid (g/L)	Succinic Acid (g/L)	Glucose + Fructose (g/L)	Glycerol (g/L)
NG-108	8,02 ± 1,34c	3,43 ± 0,06bc	5,96 ± 0,85bc	0,1 ± 0,05b	1,95 ± 0,25abc	2,04 ± 1,03b	0,58 ± 0,05ab	67,77 ± 22,01ab	3,02 ± 0,08abcd
A11-612	8,59 ± 0,87c	3,24 ± 0,07d	9,01 ± 1,34a	0,14 ± 0,03b	1,39 ± 0,47d	5,18 ± 1,48a	0,72 ± 0,03a	57,27 ± 16,45b	3,07 ± 0,09ab
MJ-311	8,27 ± 0,58c	3,42 ± 0,02bc	6,08 ± 0,07bc	0,08 ± 0,04b	2,08 ± 0,03ab	1,99 ± 0,11b	0,6 ± 0,04a	63,49 ± 8,56ab	3,22 ± 0,25abc
BD-612	7,62 ± 0,74bc	3,43 ± 0,03bc	5,68 ± 0,47bc	0,04 ± 0,02b	2,05 ± 0,06ab	1,82 ± 0,55b	0,51 ± 0,05a	76,36 ± 13,34ab	2,82 ± 0,5abcd
EM-119	7,44 ± 0,65abc	3,43 ± 0,02bc	5,79 ± 0,5bc	0,08 ± 0,04b	2,09 ± 0,11ab	1,7 ± 0,53b	0,58 ± 0,03a	77,49 ± 9,5ab	3,32 ± 0,33a
L1	7,46 ± 0,78abc	3,36 ± 0,05 cd	6,84 ± 0,77ab	0,12 ± 0,03b	1,87 ± 0,23bc	2,92 ± 1,02ab	0,59 ± 0,03a	76,14 ± 11,54ab	2,81 ± 0,18 cd
L3	7,51 ± 0,59abc	3,34 ± 0,03 cd	6,46 ± 0,6ab	0,12 ± 0,03b	2,01 ± 0,15abc	2,63 ± 0,63b	0,58 ± 0,04a	77,19 ± 10,44ab	2,81 ± 0,23abcd
Concerto	7,47 ± 0,61abc	3,49 ± 0,04ab	4,62 ± 0,11c	0,09 ± 0,03b	2,34 ± 0,15ab	0,62 ± 0,17c	0,42 ± 0,04bcd	77,45 ± 11,46ab	3,31 ± 0,31a
Excellence	8,12 ± 0,40c	3,31 ± 0,04bcd	6,92 ± 0,77ab	0,21 ± 0,02b	1,96 ± 0,18bcd	3,22 ± 0,83ab	0,56 ± 0,08abc	66,31 ± 6,61ab	2,49 ± 0,24d
Laktia	7,38 ± 1,15abc	3,46 ± 0,06ab	5,2 ± 0,49bc	0,18 ± 0,07b	2,4 ± 0,17ab	1,18 ± 0,64bc	0,58 ± 0,06ab	78,45 ± 18,21ab	3,23 ± 0,39abc
Levulia Alcomeno	6,26 ± 0,41a	3,40 ± 0,06bc	6,53 ± 0,02bc	0,12 ± 0,05b	2,04 ± 0,11bcd	2,49 ± 0,25b	0,52 ± 0,04 cd	96,16 ± 5,45a	2,65 ± 0,35d
EnartisFerm QK	6,45 ± 0,9ab	3,31 ± 0,05 cd	6,97 ± 1,41ab	0,12 ± 0,04b	1,76 ± 0,4cd	3,13 ± 1,37ab	0,65 ± 0,05a	91,3 ± 12,92ab	2,65 ± 0,02bcd
Octave	7,64 ± 0,10bc	3,50 ± 0,07ab	4,75 ± 0,25c	0,17 ± 0,04b	2,5 ± 0,12ab	0,55 ± 0,05c	0,57 ± 0,03abc	72,93 ± 1,11ab	3,36 ± 0,39abc
AWRI-796	11,26 ± 0,61d	3,56 ± 0,04a	4,47 ± 0,09c	0,55 ± 0,02a	2,48 ± 0,07a	0,03 ± 0,04d	0,3 ± 0,03d	17,28 ± 9,38c	3,68 ± 0,2a

Results are mean ± SD of three replicates. Different letters indicate statistical significance between groups.

Taking into consideration the ethanol production, despite the moderate Crabtree effect observed in this species, all the *L. thermotolerans* strains exhibited high levels of glucose and fructose in the final content, ranging from 57.27 to 96.16 g/L (A11-612 and Levulia), indicating that the inoculated strains consumed approximately 52 % to 72 % of the initial sugar concentration. In contrast, the *S. cerevisiae* control had 17.28 g/L (Table 1) of residual sugars.

Previous studies conducted by our research group have demonstrated a significant variability in ethanol production among *L. thermotolerans* strains, ranging from 4.24 % to 10.6 % (v/v) (Vicente et al., 2021). This is consistent with previous findings for the Concerto and Laktia commercial strains, which exhibited ethanol concentrations of 8 % and 6.2 % (v/v) respectively (Vaquero et al., 2020). In accordance with the results of the present study, no statistically significant differences were observed, with average ethanol concentrations of 7.47 % and 7.38 % (v/v) respectively.

Another metabolite strongly associated with sugar metabolism and alcoholic fermentation is glycerol, which exhibited variation in this study ranging from 2.49 g/L for the Excellence commercial strain to 3.36 g/L for the Octave strain. Our study demonstrated a strain variability of 26 %, which is consistent with previous findings ranging from 20 % to 50 % for *L. thermotolerans* strains (Benito, 2018, Vicente et al., 2021), as observed in *S. cerevisiae* (Benito, 2018). Several studies have reported no statistically significant differences in glycerol production among different strains. However, it is important to note that these studies employed mixed fermentations with a *S. cerevisiae* strain, making them incomparable with our results. In internal comparisons of various commercial *L. thermotolerans* strains (Laktia, Levulia, and Concerto) in sequential fermentation, no statistically significant differences were observed in the final glycerol concentration, despite variations between 6 % and 14 % (Hranilovic et al., 2021, Hranilovic et al., 2022, Snyder et al., 2021). Similar results were obtained in studies comparing other commercial strains (Excellence, Levulia, and EnartisFerm QK) (Vicente et al., 2023a). In such studies, the contribution of *L. thermotolerans* may go unnoticed due to the masking effect of *S. cerevisiae*, rendering the differences statistically insignificant.

Regarding these results, it is evident that the role of *L. thermotolerans* is influenced by both the natural matrix utilized in the fermentations and the fermentative microbial partner employed. To ensure a proper completion of alcoholic fermentation and prevent high residual sugar levels, it is necessary to combine *L. thermotolerans* with more fermentative yeast strains, such as *S. cerevisiae* or *S. pombe*. The impact of these yeasts on the fermentative process is somewhat overshadowed by the more robust fermentative yeasts they are combined with (e.g., glycerol production). Nonetheless, the role of *L. thermotolerans*, which reduces the alcohol concentration due to its alternative metabolism involving lactic acid production, has been extensively investigated when compared to *S. cerevisiae* (Hranilovic et al., 2021, Snyder et al., 2021; Vicente et al., 2023a; Vicente et al., 2023b).

### 3.2 The content and pH influence of organic acids

One of the main traits of *L. thermotolerans* is the production of lactic acid that allows the biological management of acidy, together with the decrease in the malic acid content of the resulting wines. The use of natural must in experimental designs is sometimes not adequate, since, if it is sterilized by temperature (i.e., pasteurization or autoclaving) several nutrients could be lost (e.g., amino acids or vitamins) and filtering-sterilization is sometimes difficult due to its particulate content. The indigenous bacterial population naturally present in grape musts, among them, lactic acid, and acetic acid bacteria, may influence the results regarding organic acids content. Here we employed a synthetic media, sterilized through 0.45 μm filters, eliminating any possible bacteria that could interfere with the results.

The production of lactic acid exhibits significant variability, with final contents ranging from 0.55 to 5.18 g/L for the *L. thermotolerans* strains under study (Table 1). Previous studies have reported final concentrations ranging from 0 to 12 g/L, although only a few studies have reported values higher than 6 g/L (Benito, 2018, Vicente et al., 2021). The strain Octave yielded the lowest concentration, while the autochthonous isolated strain A11-612 produced the highest. Among the commercial strains, Excellence and EnartisFerm QK exhibited the highest concentrations, with final average values of 3.22 and 3.13 g/L, respectively. Previous studies have reported varying data on commercial offerings and lactic acid production, including Excellence (2.7 g/L), EnartisFerm QK (0.8 g/L), Levulia (1.0–2.8 g/L), Laktia (1.5–5.8 g/L), and Concerto (0.5–3.4 g/L) (Hranilovic et al., 2021, Hranilovic et al., 2022, Snyder et al., 2021; Vicente et al., 2023a).

The differential production of lactic acid directly impacted the final total acidity, which ranged from 4.75 to 9.01 g/L, while the *S. cerevisiae* control exhibited a final total acidity of 4.47 g/L (Table 1). Some of the studied strains, which produced lower amounts of lactic acid, did not show statistically significant differences compared to the *S. cerevisiae* control. Previous studies have reported that *L. thermotolerans* can increase total acidity in wine conditions, ranging from values close to 0 g/L up to approximately 5 g/L, depending on the amount of lactic acid produced (Benito, 2018). The impact of lactic acid content on total acidity is closely linked to pH regulation. The *L. thermotolerans* fermentations demonstrated lower pH values than the *S. cerevisiae* controls, with differences ranging from 0.06 to 0.32 pH units.

The study utilized a synthetic matrix in which the content of malic acid was known and quantified, and no lactic acid bacteria, which consume malic acid and produce lactic acid, were present. The final concentrations of malic acid in the different *L. thermotolerans* strains studied varied in the final wines, ranging from 1.39 g/L (A11-612) to 2.5 g/L (Octave), starting from an initial concentration of 3 g/L in the synthetic grape must (SGM). This resulted in reductions ranging from 16 % to 54 % of the total initial concentration. Earlier studies have previously explored this characteristic of *L. thermotolerans*, presenting data on reductions of approximately 20 %, while a few strains were able to consume over 50 % (Blanco et al., 2020, Hranilovic et al., 2021, Vicente et al., 2021).

The parameter of succinic acid has recently become an interesting selection criterion due to its salty taste in concentrations over 100 mg/L, which has the potential to enhance the minerality character of specific wines (Baroň & Fiala, 2012). The use of this yeast species in wine fermentation can significantly impact this organoleptic characteristic, as evidenced by the observed increase in the final concentration of succinic acid. In the case of SGM, a concentration increase was observed, ranging from 0.42 g/L for Concerto to 0.72 g/L for A11-612, whereas the *S. cerevisiae* control produced 0.30 g/L. Previous studies have examined the role of *L. thermotolerans* in relation to this parameter in natural musts, observing increments ranging from 0.27 to 0.59 g/L (Benito, 2018, Binati et al., 2019, Hranilovic et al., 2021, Vicente et al., 2022).

When considering volatile acidity, it is important to take into account strains with low acetic acid production under fermentative conditions, as it is one of the main characteristics that can impact wine quality. The final concentration of acetic acid for the different *L. thermotolerans* strains studied ranged from 0.04 to 0.21 g/L, without significant statistical differences. In contrast, the *S. cerevisiae* strain exhibited higher acetic acid production, reaching up to 0.55 g/L (Table 1). These values are conducive to producing high-quality wines, as the concentration remains below the olfactory threshold of 0.8 g/L (Ruiz et al., 2019). Among the commercial strains, all demonstrated acceptable concentrations ranging from 0.09 to 0.21 g/L (Concerto and Excellence, respectively). Previous studies have reported a reduction of approximately 40 % in acetic acid content during mixed fermentations with *S. cerevisiae* (Vicente et al., 2021).

### 3.3 The impact of L. Thermotolerans on the volatile profile of wine

The production of volatile compounds, including esters, higher alcohols, and fatty acids, may play a crucial role in the interaction between species. Therefore, analyzing these compounds under single fermentation conditions, using a neutral medium such as SGM, is crucial to determine the primary volatile compounds produced by this species. Although the interactions between *L. thermotolerans* and *S. cerevisiae* in actual fermentative conditions are more complex, we conducted single fermentations of these yeast species in synthetic grape must to evaluate the impact of *L. thermotolerans* under controlled conditions. It is important to note that the impact of other species, such as *S. cerevisiae* and non-*Saccharomyces* yeasts, along with the characteristics of the must variety, can influence yeast performance and the production of various volatile compounds (Zupan et al., 2013, Avbelj et al., 2016).

The production of esters by *L. thermotolerans* is usually lower if compared to the production by *S. cerevisiae.* The reduction regarding the content in these volatile compounds is from 30 to 60 % for A11-612 and Levulia Alcomeno respectively and are driven by the decrease in the production of acetic acid ethylester (Table 2). A lower concentration of this compound is related with the general lower production of acetic acid that *L*. *thermotolerans* presents. This compound is produced both under aerobic and anaerobic conditions, using ethanol and acetate as substrates and is generally related to fruity aromas, such as pineapple or banana (Zang et al, 2020). Since *L. thermotolerans* shows a lower production of acetic acid than *S. cerevisiae*, the results agree with this observation. The reported threshold for acetic acid ethyl ester is 12 mg/L (Gómez-Míguez et al., 2007), implying an Odour Activity Value (OAV) of 8.58 units for the *S. cerevisiae* control at a concentration of 103.56 mg/L. Meanwhile, the highest OAV for *L. thermotolerans* production is 6.16, and the lowest is 3.41. This discrepancy clearly suggests that the impact of this volatile compound would be 28 % to 60 % lower in pure *L. thermotolerans* fermentations.

**Table 2:** Final volatile compound profiles of pure fermentations in Synthetic Grape Must (SGM). The Lachancea thermotolerans strains used were NG-108, A11-612, MJ-311, BD-612, EM-119, L1, L3 (Complutense University of Madrid, Madrid, Spain), Concerto (CHR Hansen, Denmark), Excellence (X'Fresh Lamothe-Abiet, France), Laktia (Lallemand, Canada), Levulia Alcomeno (AEB Group, Italy), EnartisFerm QK (Enartis, Italy) and Octave (CHR Hansen, Denmark). The Saccharomyces cerevisiae control strain used was AWRI-796 (Maurivin, Australia). Olfactory threshold of each compound is indicated (Gómez-Míguez et al., 2007).

Strain	Acetic acid ethylester [mg/L]	i-Butanol [mg/L]	Propionic acid ethylester [µg/L]	3-Methyl-butanol [mg/L]	2-Methyl-butanol [mg/L]	i-Butyric acid ethylester [µg/L]	Butyric acid ethylester [µg/L]	Lactic acid ethylester [µg/L]	Acetic acid 3-methylbutylester [µg/L]	Hexanoic acid [mg/L]	Hexanoic acid ethylester [µg/L]	2-Phenyl-ethanol [mg/L]	Octanoic acid [mg/L]	Acetic acid phenylethylester [µg/L]
**Odour threshold^1^**	12.264	150.00	–	30.00	40.00	20.00	15.00	154,636	30.00	0.42	14.00	14.00	0.5	250.00
NG-108	47.02 ± 4.38 cd	11.15 ± 1.73b	206.56 ± 14.42abc	41.86 ± 11.29ab	14.07 ± 4.18bc	71.62 ± 3.23a	93.34 ± 5.45b	61.41 ± 40.07b	153.33 ± 8.01a	3.92 ± 0bc	182.15 ± 0.31b	12.62 ± 3.34a	2.02 ± 0b	18.11 ± 0.6bc
A11-612	74.21 ± 7.45b	11.97 ± 2.96ab	235.25 ± 27.84abc	66.02 ± 14.9ab	13.59 ± 1.27abc	85.85 ± 4.03a	104.68 ± 4.01b	211.29 ± 107.99a	179.02 ± 20.94a	3.94 ± 0.01b	185.66 ± 0.25b	17.92 ± 1.69a	2.04 ± 0b	18.43 ± 0.71bc
MJ-311	47.24 ± 2.23 cd	12.02 ± 2.46ab	234.27 ± 62.46abc	64.65 ± 9.91ab	16.53 ± 2.47abc	114.6 ± 37.43a	99.84 ± 3.54b	50.6 ± 4.05b	165.37 ± 27.23a	3.92 ± 0.01bc	181.36 ± 1.26b	17.13 ± 6.62a	2.02 ± 0.01b	18.54 ± 1.11bc
BD-612	58.16 ± 4.78bcd	17.74 ± 1.9ab	221.53 ± 25.92abc	67.95 ± 8.34ab	24.13 ± 6.21a	127.42 ± 29.18a	95.34 ± 1.13b	40.49 ± 9.6b	167.46 ± 8.35a	3.91 ± 0.01bc	180.61 ± 1.66b	13.84 ± 3.94a	2.02 ± 0b	16.89 ± 0.21bc
EM-119	60.71 ± 5.01bcd	15.53 ± 1.8ab	272.12 ± 47.2ab	78.35 ± 7.72a	20.02 ± 1.09abc	103.05 ± 17.94a	96.34 ± 0.63b	47.87 ± 18.45b	205.57 ± 33.31a	3.91 ± 0bc	183.07 ± 1.61b	16.59 ± 1.79a	2.02 ± 0.01b	17.37 ± 0.9bc
L1	55.79 ± 11.05bcd	11.92 ± 1.29ab	247.62 ± 100.28abc	60.24 ± 6.48ab	16.92 ± 0.63abc	107.12 ± 11.2a	97.12 ± 1.47b	77.63 ± 43.05b	157.2 ± 19.94a	3.91 ± 0.01bc	180.95 ± 0.85b	15 ± 3.14a	2.02 ± 0.01b	15.52 ± 0.1c
L3	72.96 ± 4.8b	11.87 ± 2.62ab	257.35 ± 62.8ab	69.51 ± 11.15ab	16.85 ± 2.41abc	91.79 ± 13.47a	95.15 ± 1.81b	69.28 ± 23.04b	160.02 ± 13.89a	3.91 ± 0.01bc	181.35 ± 1.07b	15.11 ± 4.03a	2.02 ± 0.01b	16.38 ± 0.23bc
Concerto	46.77 ± 7.54 cd	17.22 ± 3.66ab	278.7 ± 53.46ab	71.7 ± 4.35ab	23.22 ± 3.05ab	127.9 ± 51.52a	107.4 ± 1.7b	21.02 ± 1.23b	182.12 ± 40.2a	3.9 ± 0.01c	181.7 ± 0.65b	15.85 ± 4.52a	2.01 ± 0.01b	17.49 ± 0.98bc
Excellence	44.57 ± 8.24 cd	11.19 ± 3.86b	129.12 ± 19.27bc	65.08 ± 22.57ab	17.87 ± 6.35abc	59.65 ± 6.06a	92.74 ± 6.19b	98.86 ± 32.25b	142.56 ± 4.59a	3.92 ± 0.01bc	179.16 ± 1.48b	16.62 ± 9.52a	2.03 ± 0b	17.2 ± 0.98bc
Laktia	61.5 ± 9.11bcd	10.32 ± 1.41b	195.74 ± 77.5abc	50.92 ± 5.79ab	11.27 ± 1.13c	108.66 ± 55.88a	94.83 ± 1.57b	40.02 ± 21.29b	182.3 ± 17.01a	3.93 ± 0.02bc	183.48 ± 2.16b	9.8 ± 0.87a	2.05 ± 0.02b	15.97 ± 0.71bc
Levulia Alcomeno	41.69 ± 5.18d	17.55 ± 3.53ab	196.3 ± 51.55abc	67.21 ± 8.93ab	20.07 ± 1.79abc	131.95 ± 30.94a	96.67 ± 2.8b	53.88 ± 9.52b	197.01 ± 33.78a	3.93 ± 0bc	183.73 ± 0.18b	14.37 ± 3.69a	2.02 ± 0.01b	17.3 ± 0.65bc
EnartisFerm QK	66.68 ± 7.87bc	17.17 ± 3.05ab	313.27 ± 18.16a	74.94 ± 26.66a	17.92 ± 2.83abc	144.98 ± 13.54a	100.46 ± 2.51b	55.7 ± 19.84b	203.36 ± 21.94a	3.91 ± 0bc	181.66 ± 1.98b	15.55 ± 4.1a	2.02 ± 0b	20.48 ± 4.49ab
Octave	48.21 ± 7.22 cd	11.95 ± 1.04ab	211.87 ± 41.64abc	64.37 ± 2.73ab	16.04 ± 1.01abc	116.9 ± 21.68a	92.12 ± 2.65b	23.58 ± 0.56b	196.39 ± 13.44a	3.92 ± 0bc	182.5 ± 1.05b	12.21 ± 0.82a	2.02 ± 0b	17.25 ± 0.13bc
AWRI-796	103.56 ± 18.4a	19.5 ± 4.23a	100.9 ± 13.37c	36.89 ± 9.41b	15.25 ± 4.87abc	119.33 ± 39.76a	169.71 ± 21.43a	18.07 ± 0.25b	195.71 ± 41.12a	4.05 ± 0.04a	287.91 ± 6.79a	6.47 ± 1.61a	2.19 ± 0.04a	23.32 ± 2.54a

Results are mean ± SD of three replicates. Different letters indicate statistical significance between groups. ^1^ Odour thresholds are in the same units as compounds.

Other interesting compounds, despite present in a lower concentration, that are usually produced in lower values by *L. thermotolerans*, if compared to *S. cerevisiae*, are: butyric acid ethylester (50 %), acetic acid phenylethylester (15 to 50 %), and hexanoic acid ethylester, an ester derived from fatty acids that is produced in around 40 to 70 % less. In all the cases, this decrease in the production of different esters are related to a lower concentration, both regarding the acetic and fatty acids concentration in fermentations carried out by *L. thermotolerans.* The reduction in the concentration of this compounds influences the aromatic profile of the resulting wines. Regarding the OAVs for the different compounds, only hexanoic acid ethylester and butyric acid ethylester are above its olfactory thresholds in all the cases. Butyric acid ethylester, shows an AOV in the *S. cerevisiae* fermentation of 11.31 compared to the in *L. thermotolerans* AOVs that vary between 6.14 and 7.16 for Octave and Concerto respectively. Despite all fermentations showing concentrations of hexanoic acid ethyl ester above the olfactory threshold (14 µg/L) (Gómez-Míguez et al., 2007), the lower production of this compound by the different *L. thermotolerans* strains impacts the OAVs in these fermentations, reducing them by up to 62 % compared to the *S. cerevisiae* control.

Other esters are produced at different concentrations by *L. thermotolerans* in single fermentations, but without statistical differences: i-butyric acid ethylester, that is differentially produced depending on the strain between an increase in around a 11 % (Levulia Alcomeno) and a reduction in around a 50 % (Excellence); or acetic acid 3-methylbutylester reduced up to a 30 % (Excellence) or increased up to around a 5 % (EM-119). On the contrary, some other esters are usually increased by *L. thermotolerans* such as propionic acid ethylester, which concentration is doubled or even tripled if compared to *S. cerevisiae*; or the lactic acid ethylester, being usually increased its concentration about 2.5 times, but with some strains producing up to 10 times more. This increase is related to the increased production of lactic acid, since this product is synthesized directly from the intracellular pool of lactic and acetic acid (Ren et al., 2020). Despite these high differences regarding lactic acid derived esters, the effect on the olfactory profile is not significative due to the high olfactory threshold that this compound has (154,636 µg/L) (Gómez-Míguez et al., 2007). *L. thermotolerans* is usually linked to an increase in esters production, being wines fermented both by *L. thermotolerans* and *S. cerevisiae* usually described as increased in the esters content (Hranilovic et al., 2021, Snyder et al., 2021, Vaquero et al., 2020, Vicente et al., 2021).

The production of acetate esters by *L. thermotolerans* is usually lower compared to that of *S. cerevisiae*. The reduction in volatile compound content ranges from 30 to 60 % for A11-612 and Levulia Alcomeno, respectively, and is driven by a decrease in the production of acetic acid ethyl ester (Table 2) with a high impact in the OAVs. The lower concentration of this compound is associated with the overall lower production of acetic acid by *L. thermotolerans*. Acetic acid ethyl ester is produced under both aerobic and anaerobic conditions, using ethanol and acetate as substrates, and is generally associated with fruity aromas, such as pineapple or banana (Zang et al., 2020). The results align with the observation that *L. thermotolerans* exhibits lower production of acetic acid compared to *S. cerevisiae*. Other interesting compounds, albeit present in lower concentrations, are typically produced at lower values by *L. thermotolerans* compared to *S. cerevisiae*. These include butyric acid ethyl ester (50 % reduction), acetic acid phenylethyl ester (15 to 50 % reduction), and hexanoic acid ethyl ester, an ester derived from fatty acids, which is produced at around 40 to 70 % less. In all cases, the decrease in the production of different esters is associated with lower concentrations of both acetic and fatty acids in fermentations carried out by *L. thermotolerans.*

*L. thermotolerans* exhibits variations in the production of other esters in single fermentations. For instance, i-butyric acid ethyl ester is differentially produced depending on the strain, with Levulia Alcomeno showing an increase of around 11 % and Excellence showing a reduction of around 50 %. Similarly, acetic acid 3-methylbutyl ester is reduced by up to 30 % in Excellence but increased by around 5 % in EM-119. On the contrary, some other esters are typically increased by *L. thermotolerans*, such as propionic acid ethyl ester, which is doubled or even tripled compared to *S. cerevisiae*. Lactic acid ethyl ester is also usually increased by about 2.5 times, with some strains producing up to 10 times more. This increase is related to the elevated production of lactic acid, as it is synthesized directly from the intracellular pool of lactic and acetic acid (Ren et al., 2020). *L. thermotolerans* is often associated with an increase in ester production, and wines fermented by both *L. thermotolerans* and *S. cerevisiae* are typically described as having an increased ester content (Hranilovic et al., 2021, Snyder et al., 2021, Vaquero et al., 2020, Vicente et al., 2021).

The statistical analysis revealed that the production of higher alcohols by *L. thermotolerans* was not significantly different from that of *S. cerevisiae*. However, an overall increase was observed, ranging from 2 % to 67 %, with strain EM-119 being the greatest producer and NG-108 being the least. Detailed analysis of specific compounds showed that 2-phenyl-ethanol was the most important fusel alcohol produced by *L. thermotolerans*, with increments ranging, depending on the strain, from approximately 1.5 to 2.5 times higher than *S. cerevisiae*. This compound is generally associated with a floral perception, often described as a rose or honey odor (Liu et al., 2019), and has been identified as one of the key molecules in yeast interactions through volatile compounds (Jagtap et al., 2020, Britton et al., 2023). Other higher alcohols produced in higher concentrations by *L. thermotolerans* included 3-methyl-butanol (increases ranging from approximately 13 % to 2 times higher) and 2-methyl-butanol (produced at a concentration approximately 60 % higher, although some strains showed slightly lower values). In contrast, the concentration of i-butanol was decreased by 10 % to 50 % compared to *S. cerevisiae*. The variation in the production of higher alcohols also impacts the olfactory profile of the resulting wines. This effect is particularly notable concerning the content of 3-methyl-butanol, with OAVs for this compound ranging from 1.39 to 2.61 in NG-108 and EM-119, respectively, compared to 1.29 OAV units in *S. cerevisiae* fermentations.

The role of this species in the production of other higher alcohols remains unclear. Several strains have shown an increase in these compounds in some experiments while exhibiting a decrease in others (Hranilovic et al., 2021, Vaquero et al., 2020; Vicente et al., 2023a). The choice of the must, combined with the *S. cerevisiae* strain in conjunction with this species, may play an important role in regulating the production of these compounds. In this study, *L. thermotolerans*, fermenting a neutral synthetic must under axenic conditions, appears to increase the content of higher alcohols compared to the *S. cerevisiae* control, despite its lower fermentative capacity. This finding should be interpreted carefully, as higher alcohols can contribute both desirable and undesirable aromas to the wine, depending on the specific odor descriptor of each compound and its olfactory threshold. Nevertheless, among several non-*Saccharomyces* species, *L. thermotolerans* seems to have the greatest impact on higher alcohols (Castrillo & Blanco, 2023).

Regarding fatty acids, *L. thermotolerans* exhibited a slight, but consistent, decrease of approximately 5 % compared to *S. cerevisiae* single fermentations. Among them, octanoic acid showed the most significant reduction of approximately 7 %, while hexanoic acid displayed reductions of around 4 %. These compounds are typically associated with cheese or rancid aromas, which are undesirable in certain wines. The impact of *L. thermotolerans* on fatty acids is still not fully understood. Some studies have reported certain increases (Shekhawat et al., 2017), but most of them agree that *L. thermotolerans* tends to decrease their concentration when co-inoculated with *S. cerevisiae* (Hranilovic et al., 2021). The commercial strains employed in this study, previously examined by different researchers, have consistently shown this trend in various matrices (such as Merlot and Tempranillo red wines) and different combinations of species (both with *S. cerevisiae* and *S. pombe*) (Hranilovic et al., 2021, Vicente et al., 2023, Vicente et al., 2023, Vicente et al., 2023, Vicente et al., 2023, Vicente et al., 2023, Vicente et al., 2023). However, it should be noted that under oxygenation conditions, the production of these compounds may increase (Shekhawat et al., 2017). The reduction in the production of fatty acids also has an impact on the olfactory profile. Despite the concentration of these compounds not falling below the olfactory threshold for each compound, significant reductions are observed in the OAVs of both hexanoic and octanoic acids: 9.64 and 4.28, respectively, in *S. cerevisiae* compared to 9.28 and 4.04, respectively, in *L. thermotolerans* fermentations.

Overall, the results corroborated the hypothesis by demonstrating substantial variability among *L. thermotolerans* strains in fermentative performance, organic acid production, and volatile compound formation, reaffirming the need for individual strain assessment for their distinct roles in winemaking. The results also show that it is possible to select strains that generate higher lactic acid than the nowadays commercial offer.

## 4 Conclusion

This study presents a comparison of several *L. thermotolerans* strains using synthetic grape juice, which can be prepared in any laboratory at any time. These results can serve as a baseline for future studies, enabling researchers and yeast manufacturers to compare their future results with newly isolated strains under reproducible conditions. A high strain variability was observed for volatile and non-volatile parameters in *L. thermotolerans*. This variability, observed not only among commercial strains but also natural isolates, explains the different performance observed under winemaking conditions. In terms of fermentative performance, a moderate capacity was observed, indicating the need for combination with a strong fermentative yeast such as *S. cerevisiae*. Certain clear patterns can be linked to the use of *L. thermotolerans* in relation to organic acids. The high production of lactic acid by some strains is of great interest as it enables pH management through biological means. Succinic acid contributes to distinct organoleptic characteristics that are valuable in certain wines, while the use of *L. thermotolerans* generally leads to a decrease in volatile acidity during wine fermentation. The impact of this yeast species on volatile compounds is challenging to summarize when describing individual compounds. However, a general conclusion can be drawn: under axenic fermentative conditions, *L. thermotolerans* is characterized by an overall increase in the content of higher alcohols, accompanied by a decrease in the content of esters and fatty acids. Understanding the influence of the environment, as well as abiotic and biotic factors, on yeast metabolism is crucial for regulating fermentative performance and the production of volatile compounds. These traits are influenced by various factors, including must variety, composition, and the naturally occurring microbial community present in natural matrices.

## CRediT authorship contribution statement

**Javier Vicente:** Writing – original draft, Validation, Supervision, Software, Methodology, Investigation, Formal analysis, Data curation. **Luka Vladic:** Formal analysis, Data curation. **Eva Navascués:** Writing – review & editing, Supervision, Project administration, Methodology, Formal analysis. **Silvia Brezina:** Formal analysis. **Antonio Santos:** Writing – review & editing, Supervision, Methodology, Investigation, Formal analysis. **Fernando Calderón:** Formal analysis. **Wendu Tesfaye:** Writing – review & editing, Formal analysis. **Domingo Marquina:** Writing – review & editing, Validation, Supervision, Resources, Project administration, Methodology, Investigation, Funding acquisition, Formal analysis, Data curation, Conceptualization. **Doris Rauhut:** Writing – review & editing, Supervision, Methodology, Formal analysis, Conceptualization. **Santiago Benito:** Writing – review & editing, Writing – original draft, Visualization, Validation, Supervision, Software, Resources, Project administration, Methodology, Investigation, Funding acquisition, Formal analysis, Data curation, Conceptualization.

## Declaration of competing interest

The authors declare the following financial interests/personal relationships which may be considered as potential competing interests: Santiago Benito reports financial support was provided by Spanish Ministry of Science and Innovation. If there are other authors, they declare that they have no known competing financial interests or personal relationships that could have appeared to influence the work reported in this paper.

## Data Availability

Data will be made available on request.
